# Association between type 2 inflammatory diseases and neurodevelopmental disorders in low-birth-weight children and adolescents

**DOI:** 10.3389/fpsyg.2024.1292071

**Published:** 2024-02-22

**Authors:** Hengye Huang, Kelvin Pengyuan Zhang, Karol Kexin Sun, Guangjun Yu

**Affiliations:** ^1^School of Public Health, Shanghai Jiao Tong University School of Medicine, Shanghai, China; ^2^Department of Epidemiology, School of Public Health, University of Michigan, Ann Arbor, MI, United States; ^3^Department of Biomedical Engineering, Whiting School of Engineering, Johns Hopkins University, Baltimore, MD, United States; ^4^Shanghai Engineering Research Center for Big Data in Pediatric Precision Medicine, Center for Biomedical Informatics, Shanghai Children's Hospital, School of Medicine, Shanghai Jiao Tong University, Shanghai, China

**Keywords:** low-birth-weight, neurodevelopmental disorders, asthma, atopic diseases, intellectual disability, autism spectrum disorder, attention deficit hyperactivity disorder, learning disability

## Abstract

**Background:**

Evidence of the association of certain neurodevelopmental disorder with specific type 2 inflammatory (T2) disease has been found. However, the association of various neurodevelopmental disorders with T2 diseases as a whole remains unclear in low-birth-weight (LBW) infants.

**Objective:**

To evaluate the association of type 2 inflammatory (T2) diseases with intellectual disability (ID), autism spectrum disorder (ASD), attention deficit hyperactivity disorder (ADHD), and learning disability (LD) in LBW children and adolescents.

**Methods:**

The study sample was derived from 2005 to 2018 National Health Interview Survey sample child files. LBW children and adolescents aged 3–17 were included. History of T2 diseases (including asthma and atopic dermatitis) and four neurodevelopmental disorders were reported by adults in families. The relationship between T2 diseases and the risk of four neurodevelopmental disorders was investigated through multiple-weighted logistic regression. Age, sex, race/ethnicity, region, highest education in family and ratio of family income to the poverty threshold were adjusted as covariates for model estimation. Subgroup analyses were conducted by age stratification (3–11 and 12–17 years), sex (male and female), and race (white and non-white).

**Results:**

11,260 LBW children aged 3–17 years [mean age (SE), 9.73 (0.05) years] were included, in which 3,191 children had T2 diseases. History of T2 diseases was associated with an increased risk of neurodevelopmental disorders, with an OR of 1.35 (95% CI, 0.99–1.84) for ID, 1.47 (95% CI, 1.05–2.05) for ASD, 1.81 (95% CI, 1.51–2.16) for ADHD, and 1.74 (95% CI, 1.49–2.04) for LD following the adjustment of all the covariates. The correlations between T2 disorders and each of the four neurodevelopmental disorders were significantly different by sex and race (all *P* for interaction < 0.001), and no differences were found in age stratification (all *P* for interaction > 0.05).

**Conclusion:**

In a nationally representative sample of children, we found a significant association of T2 diseases with ASD, ADHD, and LD, even after adjusting for demographic baseline. We also found that the association of T2 disease with neurodevelopmental disorders differed between sex and race. Further investigation is needed to evaluate causal relationships and elucidate their potential mechanisms.

## 1 Introduction

Low-birth-weight (LBW) is defined as weight at birth of < 2,500 g and remains a major public health problem globally. Approximately 15–20% of newborns are underweight worldwide and >20 million LBW infants are born each year (World Health Organization, 2014). LBW occurs because of preterm birth (before 37 completed weeks of gestation), intrauterine growth restriction, or both. Preterm birth constitutes one of the leading causes of perinatal mortality and lifelong morbidity globally (Liu et al., 2016). Premature infants are susceptible to lifelong neurological conditions, such as cerebral palsy, autism spectrum disorder (ASD), learning disability (LD), and cognition or developmental delay (Mwaniki et al., 2012; Spittle et al., 2018; Laverty et al., 2021), alongside respiratory disorders, such as asthma (Moschino et al., 2020). Likewise, the consequences of LBW include elevated fetal and neonatal mortality and morbidity, including poor cognitive development, long-term neurological disability, impaired language development and academic performance, and elevated risk of chronic diseases later in life (Risnes et al., 2011; Zerbeto et al., 2015; Yang et al., 2021). The impaired development of brain and immune system is a crucial factor in a increased risk of several diseases in LBW and preterm infants.

During fetal life, the T helper type 2 (Th2) immune response prevails. LBW infants may be at an increased risk of type 2 immune dysregulation, and consequently, type 2 inflammatory (T2) diseases owing to the underdevelopment of the immune system (Melville and Moss, 2013). Clinically, this is known as atopic diseases which includes asthma, atopic dermatitis (AD), allergic rhinitis (AR). In a birth cohort of the general population, the 12-month prevalence of any atopic disease was high (40.3%), mainly due to the high prevalence of rhinoconjunctivitis (32.8%), while asthma prevalence was 12.9% and atopic dermatitis prevalence was 8.1% (Christiansen et al., 2016). T2 diseases are attributed to the dysregulation of the immune system. Based on the similarity of the underlying immune mechanisms, children with a certain T2 disease tend to have other T2 diseases. Asthma and AD are typical diseases of over-active Th2 immune response (Akdis et al., 2020). AD (eczema) is a chronic skin disease that usually begins early in life and is generally associated with asthma and AR [American Academy of Allergy Asthma & Immunology (AAAAI), 2023]. Long-term symptoms have been observed, which may persist in adulthood or even become lifelong (Costanzo et al., 2022). Real-world evidence indicates that children with AD exhibit a higher prevalence of other T2 diseases than children without AD (Paller et al., 2022). Patients with asthma are further susceptible to comorbidities other than T2 diseases (Price et al., 2021).

Patients with T2 diseases are at an increased risk of abnormal neurological conditions. A meta-analysis reported an association of atopic disorders (including asthma, and atopic eczema) with attention deficit hyperactivity disorder (ADHD) in children and adolescents, with individuals being 30–50% tended to develop ADHD compared with controls (van der Schans et al., 2017a,b). Another study revealed a 2.7-fold increase in developmental delays in children with asthma, AD, AR, and food allergies (Jackson-Cowan et al., 2021a,b). Systematic reviews and meta-analysis have found significant association of asthma with ADHD (OR = 1.53, 95% CI, 1.41–1.65) (Cortese et al., 2018), AD with ADHD (OR = 1.28; 95% CI, 1.18–1.40), AD with ASD (OR, 1.87; 95% CI, 1.30–2.68) (Cheng et al., 2023). Furthermore, except for ASD and ADHD, a recent US longitudinal birth cohort study also reported consistent associations between various kinds of NDs and three atopic disorders (asthma, AD, and AR) (Qu et al., 2022). Mechanism studies were also in line with this idea, a review has reported that immune and nervous system are functionally linked, and immune system abnormalities impair neurodevelopment and brain function (Zengeler and Lukens, 2021).

Although there has been some research regarding the association of asthma or AD with neurodevelopmental delays, neurological conditions, and emotional disorders, relevant studies and data are limited, particularly for children. Furthermore, few studies have combined different kinds of T2 diseases and done analysis regarding the whole. Thus, this study used nationally representative data of United States (US) children to verify the association between T2 diseases, including asthma or AD, and four common neurodevelopmental disorders, such as intellectual disability (ID, also known as mental retardation or developmental delays), ASD, ADHD, and LD. Owing to the lack of preterm birth-related information in this database, LBW infants were selected as the study population for majority of them were preterm infants. We have not seen any studies using such a population to investigate this relationship among LBW infants.

## 2 Materials and methods

### 2.1 Data sources and study sample

The data used in this study were derived from the National Health Interview Survey (NHIS), a national health survey conducted by the National Center for Health Statistics (NCHS) since 1957 (National Center for Health Statistics (NCHS), 2022). NHIS utilizes geographic cluster sampling technology to continuously select the representative housing unit samples in the country for cross-sectional survey. By completing interviews in the homes of the respondents or via telephone, NHIS obtained and evaluated a wide range of health data to monitor health status of the non-institutionalized civilian population in the US. As no variables were present regarding birth weight and skin allergy in 2019 and 2020, we downloaded study-related data spanning 2005–2018 from the NHIS Data, Questionnaires, and Related Documentation module on the official website of NHIS (sample size = 155,902). Then we analyzed LBW subjects aged 3–17 years in the NHIS Sample Child File (sample size = 128,815). From 2005 to 2018, the overall response rate of the family module ranged from 63.4 to 87.0%, with the response rate for the child module ranging from 59.2 to 78.8%. The children lacking data on anyone of asthma, eczema or any type of skin allergy, ID, ASD, ADHD, or LD, were excluded. The final eligible sample consisted of 11,260 subjects. The NHIS was approved by the Research Ethics Review Board of the NCHS and informed consent was obtained from all participants (National Center for Health Statistics, 2019). This study was conducted and reported in accordance with the STROBE reporting guidelines.

### 2.2 Variables and covariates

In the NHIS interview, information regarding the sample child is collected from an adult in the family who has the most knowledge about the health of the child. According to relevant literature, we defined eczema or any kind of skin allergy in NHIS as AD (Hou and Silverberg, 2022; Xu et al., 2022). Because of lacking information on AR in NHIS, we defined the occurrence of asthma or AD as a T2 disease. Main outcomes of neurodevelopmental disorders are designated by ID, ASD, ADHD, and LD. Information about historic diagnosis of these six diseases were determined by adults' responses to the questions listed in eTable 1. We selected age, sex, race/ethnicity, region, highest education in family and ratio of family income to the poverty threshold as covariates for model estimation. Basic information was collected using a standard questionnaire or protocol. Information regarding age, sex, race/ethnicity, highest education in family, and ratio of family income to the poverty threshold was collected as family core data, which was reported by an adult in the family. The region was acquired from Unit Control File and was divided into Northeast, Midwest, South, and West. Race/ethnicity comprised White people, Black/African Americans, American Indians and Alaska Natives (AIAN), Asians, and other races. Highest education in family was classified into three groups: (i) less than high school, (ii) high school, and (iii) college and higher. The ratio of family income to the poverty threshold was categorized into four groups: (i) < 1, (ii) ≥1 and < 2, (iii) ≥2 and < 4, and (iv) ≥4.

### 2.3 Statistical analysis

By introducing the official NHIS published sampling weights, strata, and primary sampling units into data analysis, the NHIS database can be widely used to estimate the prevalence of a wide range of diseases nationwide in the United States (National Center for Health Statistics, 2019). The numeric and categorial variables were statistically described using “mean (standard error)” and “percentage [95% confidence intervals (Cis)]”, respectively. *T*-tests and Chi-square tests were employed to compare the prevalence of diseases in different groups. Weighted multiple logistic regression was used to estimate the adjusted odds ratios (ORs, 95% CIs) and corresponding *P*-values of neurodevelopmental disorders according to the presence of asthma, AD and T2 diseases. Age and sex were adjusted routinely in Model 1. Race/ethnicity, region, highest education in family and ratio of family income to the poverty threshold were additionally adjusted in Model 2. We conducted subgroup analyses and calculated *p*-values for interactions by age (3–11 and 12–17 years), sex (male and female) and race/ethnicity (white and non-white) to evaluate potential effect modification (Yang et al., 2021). Data were analyzed using SAS version 9.4 (SAS Institute, NC). Statistical significance was considered with a two-sided *P*-value of < 0.05.

## 3 Results

There were 11,260 children and adolescents (aged 3–17 years) included with a mean age of 9.73 (0.05) years in our study [47.3%, boys (95% CI, 46.1–48.5%) and 52.7% girls (95% CI, 51.5–53.9%)]. The racial composition of the cohort was 67.3% (95% CI, 65.9–68.6%) White people, 21.9% (95% CI, 20.7–23.2%) African American, 1.0% (95% CI, 0.7–1.2%) AIAN, 5.6% (95% CI, 5.0–6.1%) Asian, and 4.3% (95% CI, 3.8–4.8%) others. The study included 3,191 children with T2 diseases (prevalence = 28.3%). Among them, 2,308 had asthma (prevalence = 20.5%), and 1,376 experienced AD (prevalence = 12.2%). In the 11,260 LBW children, the weighted prevalence of ID was 2.2% (95% CI, 1.9–2.5%); that of ASD was 1.9% (95% CI, 1.6–2.2%); that of ADHD was 10.8% (95% CI, 10.1–11.5%, and that of LD was 11.7% (95% CI, 10.9–12.5%).

Male children were more likely to have asthma (*P* < 0.001). Black/African American, AIAN and other races/ethnicities appeared to have a higher prevalence of asthma than White people and Asian (*P* < 0.001). Those with a lower educational level family and with a lower ratio of family income to poverty level were more likely to have asthma than other groups (*P* < 0.001) (Table 1). Black/African American, AIAN and other races/ethnicities appeared to have a higher prevalence of AD than White people and Asian (*P* < 0.001). Those with a higher educational level family were more likely to have AD than other groups (P = 0.014) (Table 2). Male children were more likely to have T2 diseases (*P* < 0.001). Black/African American, AIAN and other races/ethnicities appeared to have a higher prevalence of T2 diseases than White people and Asian (*P* < 0.001). Those with a lower ratio of family income to poverty level were more likely to have T2 diseases than other groups (*P* < 0.001) (Table 3).

**Table 1:** Characteristics of the NHIS study participants aged 3–17 years with and without asthma 2005–2018.

**Variables**	**Overall (*N* = 11,260)**	**Children with asthma (*N* = 2,308)**	**Children without asthma (*N* = 8,952)**	***P***
Age, y, mean (SE)	9.73 (0.05)	10.26 (0.12)	9.61 (0.06)	< 0.001
**Sex, % (95% CI)**				< 0.001
Male	47.3 (46.1, 48.5)	54.5 (51.8, 57.2)	45.5 (44.2, 46.9)	
Female	52.7 (51.5, 53.9)	45.5 (42.9, 48.2)	54.5 (53.1, 55.8)	
**Race/ethnicity, % (95% CI)**				< 0.001
White people only	67.3 (65.9, 68.6)	58.9 (56.3, 61.6)	69.3 (67.8, 70.7)	
Black/African American only	21.9 (20.7, 23.2)	30.3 (27.8, 32.9)	19.9 (18.6, 21.2)	
AIAN only	1.0 (0.7, 1.2)	1.1 (0.5, 1.7)	0.9 (0.7, 1.2)	
Asian only	5.6 (5.0, 6.1)	3.6 (2.6, 4.6)	6.1 (5.4, 6.7)	
Other	4.3 (3.8, 4.8)	6.1 (4.9, 7.3)	3.8 (3.3, 4.4)	
**Region, % (95% CI)**				0.004
Northeast	16.6 (15.4, 17.8)	18.9 (16.7, 21.1)	16.0 (14.6, 17.5)	
Midwest	22.6 (20.8, 24.5)	21.8 (18.6, 25.1)	22.8 (21.0, 24.6)	
South	39.3 (37.5, 41.2)	41.2 (38.2, 44.2)	38.9 (36.9, 40.9)	
West	21.5 (20.2, 22.7)	18.1 (16.1, 20.0)	22.3 (20.9, 23.6)	
**Highest education in family, % (95% CI)**				< 0.001
Less than high school	13.7 (12.9, 14.6)	15.2 (13.1, 17.3)	13.4 (12.5, 14.3)	
High school	37.2 (36.1, 38.4)	40.8 (38.1, 43.4)	36.4 (35.0, 37.7)	
College and higher	49.1 (47.8, 50.3)	44.1 (41.2, 46.9)	50.3 (48.8, 51.7)	
**Ratio of the family income to the federal poverty threshold, % (95% CI)**				< 0.001
< 1.0	23.3 (22.1, 24.4)	26.2 (23.8, 28.6)	22.6 (21.3, 23.8)	
1.0–1.9	24.2 (23.1, 25.4)	27.8 (25.1, 30.4)	23.4 (22.1, 24.6)	
2.0–3.9	27.9 (26.7, 29.0)	26.7 (24.4, 29.0)	28.1 (26.9, 29.4)	
≥4.0	24.7 (23.4, 25.9)	19.4 (17.3, 21.5)	25.9 (24.6, 27.3)	

Data are given as weighted mean (SE) for continuous variables and weighted percentage for categorical variables. SE, standard error; AIAN, American Indians and Alaska Natives.

**Table 2:** Characteristics of participants with and without AD in the NHIS 2005–2018.

**Variables**	**Overall (*N* = 11,260)**	**Children with AD (*N* = 1,376)**	**Children without AD (*N* = 9,884)**	***P***
Age, y, mean (SE)	9.73 (0.05)	9.13 (0.14)	9.82 (0.06)	< 0.001
**Sex, % (95% CI)**				0.115
Male	47.3 (46.1, 48.5)	49.7 (46.4, 53.0)	46.9 (45.7, 48.2)	
Female	52.7 (51.5, 53.9)	50.3 (47.0, 53.6)	53.1 (51.8, 54.3)	
**Race/ethnicity, % (95% CI)**				< 0.001
White people only	67.3 (65.9, 68.6)	57.8 (54.4, 61.3)	68.6 (67.2, 70.0)	
Black/African American only	21.9 (20.7, 23.2)	30.7 (27.6, 33.9)	20.7 (19.4, 22.0)	
AIAN only	1.0 (0.7, 1.2)	1.0 (0.4, 1.5)	1.0 (0.7, 1.2)	
Asian only	5.6 (5.0, 6.1)	5.2 (3.7, 6.8)	5.6 (5.0, 6.2)	
Other	4.3 (3.8, 4.8)	5.3 (3.9, 6.7)	4.1 (3.6, 4.6)	
**Region, % (95% CI)**				0.317
Northeast	16.6 (15.4, 17.8)	15.6 (13.0, 18.1)	16.7 (15.5, 18.0)	
Midwest	22.6 (20.8, 24.5)	21.6 (18.1, 25.1)	22.8 (20.9, 24.7)	
South	39.3 (37.5, 41.2)	42.4 (38.6, 46.3)	38.9 (37.0, 40.8)	
West	21.5 (20.2, 22.7)	20.5 (17.8, 23.1)	21.6 (20.3, 22.8)	
**Highest education level in the family, % (95% CI)**				0.014
Less than high school	13.7 (12.9, 14.6)	10.8 (8.7, 13.0)	14.1 (13.2, 15.0)	
High school	37.2 (36.1, 38.4)	36.9 (34.0, 39.8)	37.3 (36.1, 38.5)	
College and higher	49.1 (47.8, 50.3)	52.3 (49.0, 55.6)	48.6 (47.3, 49.9)	
**Ratio of the family income to the federal poverty threshold, % (95% CI)**				0.738
< 1.0	23.3 (22.1, 24.4)	22.8 (19.7, 25.9)	23.3 (22.1, 24.6)	
1.0–1.9	24.2 (23.1, 25.4)	25.8 (22.8, 28.8)	24.0 (22.8, 25.2)	
2.0–3.9	27.9 (26.7, 29.0)	27.3 (24.3, 30.2)	27.9 (26.7, 29.1)	
≥4.0	24.7 (23.4, 25.9)	24.2 (21.3, 27.1)	24.7 (23.5, 26.0.)	

Data are given as weighted mean (SE) for continuous variables and weighted percentage for categorical variables. SE, standard error; AIAN, American Indians and Alaska Natives.

**Table 3:** Characteristics of participants with and without T2 diseases in the NHIS 2005–2018.

**Variables**	**Overall (*N* = 11,260)**	**Children with T2 diseases (*N* = 3,191)**	**Children without T2 diseases (*N* = 8,069)**	***P***
Age, y, mean (SE)	9.73 (0.05)	9.86 (0.10)	9.69 (0.06)	0.019
**Sex, % (95% CI)**				< 0.001
Male	47.3 (46.1, 48.5)	52.4 (50.1, 54.6)	45.3 (44.0, 46.7)	
Female	52.7 (51.5, 53.9)	47.6 (45.4, 49.9)	54.7 (53.3, 56.0)	
**Race/ethnicity, % (95% CI)**				< 0.001
White people only	67.3 (65.9, 68.6)	60.2 (57.9, 62.5)	69.9 (68.5, 71.4)	
Black/African American only	21.9 (20.7, 23.2)	28.6 (26.5, 30.8)	19.4 (18.0, 20.7)	
AIAN only	1.0 (0.7, 1.2)	1.0 (0.6, 1.5)	0.9 (0.7, 1.2)	
Asian only	5.6 (5.0, 6.1)	4.5 (3.6, 5.4)	6.0 (5.3, 6.7)	
Other	4.3 (3.8, 4.8)	5.7 (4.7, 6.6)	3.8 (3.2, 4.3)	
**Region, % (95% CI)**				0.059
Northeast	16.6 (15.4, 17.8)	17.8 (15.9, 19.6)	16.2 (14.7, 17.7)	
Midwest	22.6 (20.8, 24.5)	21.6 (18.7, 24.4)	23.0 (21.1, 24.9)	
South	39.3 (37.5, 41.2)	41.1 (38.3, 43.9)	38.7 (36.6, 40.7)	
West	21.5 (20.2, 22.7)	19.6 (17.8, 21.4)	22.2 (20.8, 23.5)	
**Highest education level in the family, % (95% CI)**				0.074
Less than high school	13.7 (12.9, 14.6)	13.1 (11.5, 14.7)	14.0 (13.0, 14.9)	
High school	37.2 (36.1, 38.4)	39.5 (37.3, 41.6)	36.4 (35.0, 37.8)	
College and higher	49.1 (47.8, 50.3)	47.5 (45.1, 49.8)	49.7 (48.2, 51.2)	
**Ratio of the family income to the federal poverty threshold, % (95% CI)**				< 0.001
< 1.0	23.3 (22.1, 24.4)	24.6 (22.5, 26.7)	22.8 (21.4, 24.1)	
1.0–1.9	24.2 (23.1, 25.4)	26.6 (24.3, 28.8)	23.3 (22.1, 24.6)	
2.0–3.9	27.9 (26.7, 29.0)	27.2 (25.2, 29.1)	28.1 (26.8, 29.5)	
≥4.0	24.7 (23.4, 25.9)	21.7 (19.8, 23.6)	25.8 (24.4, 27.2)	

Data are given as weighted mean (SE) for continuous variables and weighted percentage for categorical variables. SE, standard error; AIAN, American Indians and Alaska Natives; T2 diseases, type 2 inflammatory diseases (including asthma or/and atopic dermatitis here).

**Figure 1:**
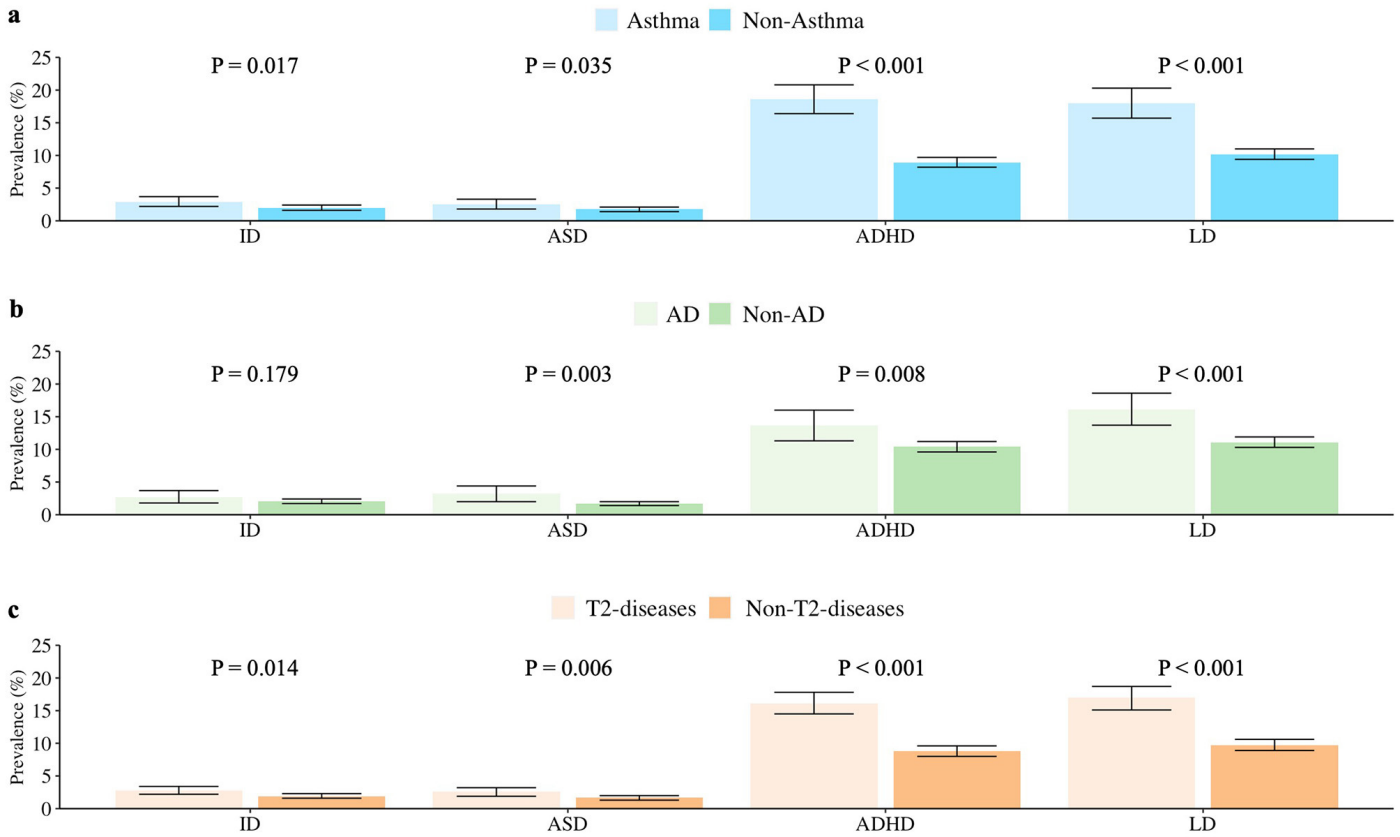
Prevalence of ID, ASD, ADHD, and LD in US children with and without **(a)** asthma **(b)** AD **(c)** T2 diseases (aged 3–17 years, NHIS 2005–2018). Data Source: National Health Interview Survey (NHIS), 2005–2018. ID, intellectual disability; ASD, autism spectrum disorder; ADHD, attention deficit hyperactivity disorder; LD, learning disability; AD, atopic dermatitis; T2 diseases, type 2 inflammatory diseases (including asthma or/and atopic dermatitis here).

Children with asthma exhibited a significantly higher prevalence of the four neurodevelopmental disorders compared with those without asthma. Among them, the children with asthma had a significantly higher risk of ID (*P* = 0.017), ASD (*P* = 0.035), ADHD (*P* < 0.001), and LD (*P* < 0.001). Children with AD or AD history exhibited higher risks of neurodevelopmental disorders than those without AD or AD history. Among them, the children with AD had a significantly higher risk of ASD (*P* = 0.003), ADHD (*P* = 0.008), and LD (*P* < 0.001). For further analysis, when asthma and AD were combined as representatives of T2 diseases, children with T2 diseases exhibited higher risks of all the four neurodevelopmental disorders than children without T2 diseases. Among them, the children with T2 had a significantly higher risk of ID (*P* = 0.014), ASD (*P* = 0.006), ADHD (*P* < 0.001), and LD (*P* < 0.001) (Figure 1).

**Figure 2:**
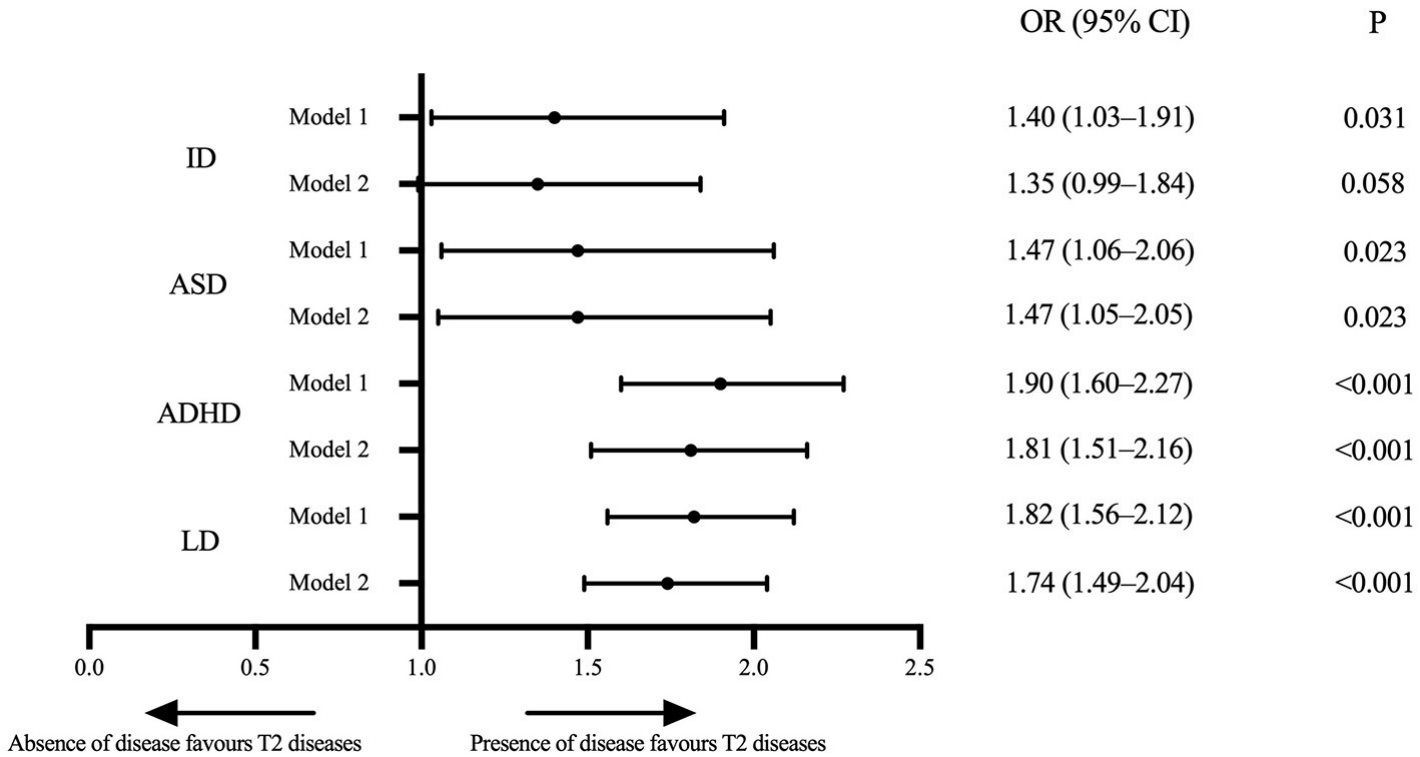
Forest plot of associations between the T2 diseases and ID, ASD, ADHD, and LD in US children aged 3–17 years in the NHIS 2005–2018. Data Source: National Health Interview Survey (NHIS), 2005–2018. Model 1: adjusted for age and sex. Model 2: adjusted for age, sex, race/ethnicity, region, the highest education level in the family and ratio of family income to the federal poverty threshold. ID, intellectual disability; ASD, autism spectrum disorder; ADHD, attention deficit hyperactivity disorder; LD, learning disability; OR, odds ratio; T2 diseases, type 2 inflammatory diseases (including asthma or/and atopic dermatitis here).

The results indicated a significant association between T2 diseases and four examined neurodevelopmental disorders, which suggested that T2 diseases are associated with higher risks of neurodevelopment deficiency. Compared to children without T2 diseases, the ORs for neurodevelopmental disorders in children with T2 diseases were 1.40 (95% CI, 1.03–1.91; *P* = 0.031) for ID, 1.47 (95% CI, 1.06–2.06; *P* = 0.023) for ASD, 1.90 (95% CI, 1.60–2.27; *P* < 0.001) for ADHD, and 1.82 (95% CI, 1.56–2.12; *P* < 0.001) for LD after adjusting age and sex. This correlation did not change markedly after additionally adjusted race/ethnicity, region, highest education in family and ratio of family income to the federal poverty level, with ORs of 1.35 (95% CI, 0.99–1.84; *P* = 0.058) for ID, 1.47 (95% CI, 1.05–2.05; *P* = 0.023) for ASD, 1.81 (95% CI, 1.51–2.16; *P* < 0.001) for ADHD, and 1.74 (95% CI, 1.49–2.04; *P* < 0.001) for LD (Figure 2).

A significant association between T2 diseases and neurodevelopmental disorders was found in most stratified population analyses. In the age stratum, adolescents aged 12–17 years with T2 diseases tended to develop ID (OR, 1.68; 95% CI, 1.10–2.56), and children aged 3–11 years with T2 diseases were more likely to develop ASD (OR,1.79; 95%CI, 1.18–2.71). However, overall, no differences were observed between the two age strata in the association between T2 diseases and four examined neurodevelopmental disorders (all P for interaction > 0.05) (eTable 2). T2 diseases were significantly correlated with ADHD (OR, 2.39; 95% CI, 1.76–3.23) and LD (OR, 1.68; 95% CI, 1.29–2.19) in girls, and significantly correlated with ID (OR, 1.58; 95%CI, 1.03–2.43), ADHD (OR, 1.54; 95%CI, 1.27–1.88), and LD (OR, 1.82; 95%CI, 1.51–2.19) in boys. Girls significantly differed from boys in the association of T2 diseases with examined four neurodevelopmental disorders (all P for interaction < 0.001) (eTable 3). Among races, the association of T2 diseases with ADHD or LD seemed stronger among non-white people than among white people (all *P* for interaction < 0.001) (eTable 4).

## 4 Discussion

We found strong association of T2 diseases with multiple neurodevelopmental disorders in this cross-sectional study of LBW children and adolescents from across the US. The prevalence of all four neurodevelopmental disorders (ID, ASD, ADHD, and LD) was higher in children with asthma or AD. The correlation was more significant between T2 diseases (namely, combining asthma and AD as a whole) and these four neurodevelopmental disorders. After adjusted age, sex, race/ethnicity, geographic region, education and economic level, T2 diseases significantly increased the risk of ASD (OR, 1.47; 95% CI, 1.05–2.05; *P* = 0.023), ADHD (OR, 1.81; 95% CI, 1.51–2.16; *P* < 0.001), and LD (OR, 1.74; 95% CI, 1.49–2.04; *P* < 0.001). Furthermore, we provided preliminarily evidence regarding a positive association of a borderline effect between T2 diseases and ID (OR, 1.35; 95% CI, 0.99–1.84; *P* = 0.058). Currently, there's limited study which combine different kinds of atopic disorders as a whole category when considering the association of atopic disorders with neurodevelopmental disorders, especially those investigated the association among large samples of children. We found a consistent positive association of T2 diseases (either asthma or AD) with four neurodevelopmental disorders in children. This result further indicates a general association between Th2 immune abnormalities and multiple neurodevelopmental disorders. The elevated risk of T2 diseases in LBW or preterm infants in addition to impaired neurodevelopment may have a unifying underlying mechanism. Clarifying this mechanism may help develop clinical disease prevention and treatment modalities.

The etiology of LBW is due to a complex interplay of numerous physical and environmental factors (Hou and Silverberg, 2022), and reduced gestational age and birth weight increases the likelihood of underdeveloped immune system (Miller et al., 2016). Cytokines associated with pregnancy can generally be divided into two categories, in one type, elevated cell-mediated immunity (Th1-type cytokines) harms the pregnancy, and in another type, suppressed strong cellular responses (Th2-type cytokines) benefit the pregnancy. During fetal life, cytokine responses are likely to favor the Th2 phenotype. Th2-bias is considered a measure to avoid the rejection of the fetus by the maternal immune system and to favor a normal pregnancy outcome (Szekeres-Bartho, 2002). Thus, normal intrauterine development largely relies on cytokine homeostasis. To some extent, LBW or prematurity is associated with impaired Th2 homeostasis. Development of immune system in fetus is completed primarily in late gestation, which impairs the immune function of LBW or preterm infants following birth. Thus, these infants exhibit defects not only in innate and adaptive immunity but also in the interaction between these two systems (Strunk et al., 2011). A review suggested that LBW is associated with an elevated risk of asthma in children and adults (Mu et al., 2014). Another study found that LBW and prematurity were risk factors for asthma and AD (Steffensen et al., 2000). Not coincidentally, the cerebral vasculature of developing children is more susceptible to infiltration by peripheral cytokines due to particular fragility, especially in LBW or preterm infants with poor developmental status (Bilbo and Schwarz, 2012). In the inflammatory state, microglia or astrocytes reduce neuronal connectivity, disrupt neurogenesis, alter the blood–brain barrier, and enhance inflammatory infiltration (Smith et al., 2014). While the vast majority of astrocytes are synthesized in the first month of life, the normal development of the brain depends on their presence (Farhy-Tselnicker and Allen, 2018). This is especially critical for LBW or premature infants with immune challenges early in life, especially with regard to the critical areas of learning and memory, such as the hippocampus.

T2 is an inflammation predominantly mediated by Th2 cells, type 2 intrinsic lymph-like cells, and associated cytokines. Atopic diseases are a group of disorders associated with the coexistence of Th2 sensitization. In a birth cohort study, 60% of children with at least one atopic disease were sensitized, while only 16% of children without atopic diseases were sensitized (Christiansen et al., 2016). Systemic inflammation due to Th2 sensitization can exert a negative impact on brain health and neurodevelopment (Bilbo and Schwarz, 2012). In children with AD or asthma, the skin epithelium and/or airways form abnormally tight junctions for relief from external irritants, which in turn induce a hypersensitive immune response (Chuang et al., 2022). The initial phase of this type of immune response is considered to be mainly mediated by Th2 cells and is characterized by elevated levels of cytokines, such as interleukin (IL)4, IL5, IL13, and immunoglobulin E (Kaas et al., 2021). Such inflammatory factors may influence neural activity in the prefrontal cortex and anterior cingulate cortex, both of which are associated with deficits in attentional control, decision making, memory, and motor output. These deficits are also considered the major symptoms of ADHD (Xu et al., 2022). Immune dysregulation has been associated with ASD diagnosis (Kim et al., 2022). Social behavior, immune function, and dopaminergic signaling are linked to adolescents' development neurologically and behaviorally (Kopec et al., 2019). AD is related to an elevated risk of comorbidities, including asthma and mental health disorders. Its pathophysiology is complex, involving strong genetic predisposition, epidermal dysfunction, and T cell–driven inflammation, with predominant type 2 mechanisms (Langan and Weidinger, 2020).

Immune system dysfunction and inflammatory processes have been considered to be potential etiological mechanisms for neurodevelopmental disorders (Bilbo et al., 2018). The immune system partakes in regulating critical early brain development, such as promoting myelin formation, synaptogenesis, and synaptic development (Hughes, 2021). Furthermore, it is involved in brain plasticity and pathogenesis of various neurological conditions, such as epilepsy, schizophrenia, and virus-induced cognitive deficits (Parker et al., 2022). Studies (Wan et al., 2020; Qu et al., 2022) also suggested that more attention should be paid in diagnosing atopic disorders in children with other types of neurodevelopmental disorders. A study indicated that the prevalence of LD is higher in children with AD, and does not rely on sociodemographic characteristics and comorbidities, such as ADHD and other atopic disorders (Wan et al., 2020). Another study revealed a 2.7-fold increase in developmental delays in children with asthma, AD, AR, and food allergies (Jackson-Cowan et al., 2021a,b). Furthermore, studies have reported an association of elevated risk of neurodevelopmental disorders in the offspring with atopic diseases in the mother as well as maternal inflammatory events induced by environmental factors during pregnancy (Bilbo et al., 2018; Croen et al., 2019).

Generally, immune disorders occur earlier than neurodevelopmental disorders. Some researchers have suggested that this correlation may be directional, in that atopic disorders usually precede neurodevelopmental disorders (van der Schans et al., 2017a,b). However, Other researchers have argued that this relationship is bilateral (Fluegge and Fluegge, 2018). Recently, a meta-analysis confirmed that asthma is a frequent comorbidity of AD (Ravnborg et al., 2021). However, AD usually presents early in life and often precedes other allergic diseases including asthma or AR. Such individuals typically have co-occurring risks that are triggered by genes affecting skin barrier function or the immune system (Nutten, 2015). A recent review has proposed new insights into the skin-brain-neural developmental connection, suggesting that epidermal and neural tissues are mediated by common prenatal developmental origins and genetic susceptibility variants (Jameson et al., 2023). Initial skin barrier integrity after birth may contribute to the integrity of the cerebral cortex and early childhood neurodevelopment. Thus, the initial postnatal skin barrier integrity may provide a helpful marker for cerebral cortex integrity and risk of early childhood neurodevelopmental delay. There may also be a common cause or a bidirectional correlation for atopic diseases and neurodevelopment disorders in LBW or preterm infants.

In addition to the neuroimmune pathway, the role of psychosocialization should also be considered. Atopic diseases not only cause physical discomfort to the child, but also bring psychological stress to the child as well as the primary caregiver (Chuang et al., 2022). Chronic stress can lead to stress sensitization. Stress in early childhood is considered to interfere with the balance between neurotransmitters and the neuroendocrine system, including the dysregulation of norepinephrine, dopamine, and the thalamic–pituitary–adrenal (HPA) axis (Nicolaides et al., 2015). The dysregulation of the HPA axis leads to delayed brain development and Th1/Th2 imbalance, thereby contributing to the immune profile of atopic diseases. The abnormal regulation of the HPA axis causing abnormal cortisol responses can affect executive function and attention in children (Van West et al., 2009). Similarly, the dysregulation of the HPA axis due to maternal stress can cause delayed brain development and Th1/Th2 imbalance in addition to immune features of atopic diseases (Van den Bergh et al., 2020). Additionally, Th2 sensitization in children causes inflammation in brain, thereby disrupting connectivity and transmission at critical developmental time points and resulting in developmental and psychological consequences (Jackson-Cowan et al., 2021a,b).

In terms of sex stratification, previous studies have reported that the immune system exhibits varying effects on neurodevelopment in both sexes. Sex hormones play important roles in allergic inflammation, estrogen as an immune enhancer and androgen as a suppressor (Taneja, 2018). These differences in immune responses can lead to differences in disease phenotypes, with autoimmunity occurring more often in females and cancer occurring more often in males (Taneja, 2018). Likewise, there are distinctions in the characteristics of neurodevelopmental processes between men and women. The male-specific developmental processes comprise cell and axon growth, synaptic assembly and activity, and energy metabolism; the female-specific processes involve cell polarity and differentiation, transcriptional repression, steroid hormones, and immune signaling (Friedrich et al., 2022). Additionally, the immune system partakes in the sex-specific regulation of neurological functions, such as the regulation of the hypothalamic–pituitary–gonadal axis development across the sexes during puberty (Granata et al., 2022). In recent years, one study reported sex differences as a prominent feature of neurodevelopmental disorders. It found that the two most common neurodevelopmental disorders (ASD and ADHD) have the male sex bias in prevalence and sex differences in symptoms (Breach and Lenz, 2023). Another study explored the underlying mechanisms by which sex differences in neurodevelopmental disorders (male sex bias) may be mediated by factors such as the protective effects of estrogen, chromosomal abnormalities, maternal immune activation, altered neurotransmission, and by neuroinflammation (Santos et al., 2022). Our results found that the correlation between T2 diseases and neurodevelopmental disorders differed significantly by sex, but the sex trend may not be the same for different developmental disorders. This may be related to the fact that immune-neural interactions have different mechanisms of action at different stages of puberty. There is no sex difference in HPA axis reactivity before puberty, but there are species-specific age and sex differences in HPA axis stress reactivity and responsiveness during puberty and mid-adolescence, and post-pubertal adolescence has been shown to be a unique period of HPA axis activity (Granata et al., 2022). Future studies could explore the differences in immune-neural interactions, and underlying mechanisms, in children and adolescents at different pubertal stages.

In terms of age stratification, neurodevelopmental disorders are characterized by different age levels. ID is a disorder that has a higher prevalence in older children as they get older and experience increased academic stress (Maulik et al., 2011). ASD, on the other hand, is a disorder with a higher prevalence in younger children, with a median first diagnosis of 5–6 years of age (Jo et al., 2015). In terms of race/ethnicity stratification, black and Hispanic children were less likely to receive joint monitoring and screening than white children (Barnard-Brak et al., 2021). This resulted in the fact that non-white children with developmental disorders were often identified later than white children and therefore missed opportunities for early intervention. Therefore, it may lead to a higher prevalence of co-morbidity between immune system diseases and neurodevelopmental disorders in non-white children.

There are several limitations to this study. First, the study lacked a specific longitudinal temporal relationship between the diagnosis of T2 diseases and that of neurodevelopmental disorders. Second, this investigation did not consider maternal perinatal disorders and lacked records of preterm birth time. The mechanisms underlying the association of T2 diseases with neurodevelopmental disorders in LBW and preterm infant populations can be further characterized in the future, alongside the association with maternal atopic diseases. Finally, the diagnostic details of the diseases were unclear, for example, those of AD. Only asthma and AD were defined as T2, with a lack of documentation of other T2 disorders such as common AR. Also, possible risk factors associated with T2 diseases, such as allergens, air pollution, and climates (Nutten, 2015; Miller et al., 2021), were not fully considered. A major strength of this study was the use of a large nationally representative sample of data, which makes the findings more stable and credible. However, the data was derived only from US and its population representation remained limited.

This study examined the association between T2 diseases and four neurodevelopmental disorders based on the LBW population. Not only because LBW is a pediatric subgroup requiring special clinical attention, but also because of the possible biological mechanisms between fetal dysplasia and altered brain development. Future studies could further explore the causal relationship between immune system diseases and neurodevelopmental disorders by incorporating age-specific and sex dimorphism, as well as neuroimmune interactions or immune-HPA interactions at different pubertal stages. The ultimate goal is to prevent the development of such diseases or co-morbidities by focusing on the holistic management of immune system diseases and associated risk factors in the infant's early life and to improve the prognosis of neurodevelopmental disorders through early intervention.

In conclusion, we found significant association of T2 diseases with ASD, ADHD, and LD in a nationally representative sample of US LBW children, as well as a borderline effect association with ID. Atopic diseases and neurodevelopmental disorders may share common responsible factors and similar pathogenesis. The association between T2 diseases and neurodevelopmental disorders differed between sex and race. However, further longitudinal studies are warranted to evaluate the causality and underlying mechanisms.

## Data availability statement

The original contributions presented in the study are included in the article/Supplementary material, further inquiries can be directed to the corresponding author.

## Ethics statement

The studies involving humans were approved by the Research Ethics Review Board of the NCHS. The studies were conducted in accordance with the local legislation and institutional requirements. Written informed consent for participation in this study was provided by the participants' legal guardians/next of kin.

## Author contributions

HH: Conceptualization, Data curation, Formal analysis, Methodology, Writing—original draft, Writing—review & editing. KZ: Data curation, Formal analysis, Methodology, Writing—original draft. KS: Visualization, Writing—original draft, Writing—review & editing. GY: Funding acquisition, Project administration, Supervision, Writing—review & editing.
