# Supplementary material for: Association between type 2 inflammatory diseases and neurodevelopmental disorders in low-birth-weight children and adolescents
Source: Front Psychol. 2024 Feb 22;15:1292071. doi: 10.3389/fpsyg.2024.1292071 (PMC10918750; doi:10.3389/fpsyg.2024.1292071)
Supplement: Supplementary file 1 [file Data_Sheet_1.docx]

**Supplement**

**eTable 1 Questions asked by the NHIS regarding the T2 diseases and neurodevelopmental abnormalities from 2005 to 2018.**

| **Variables** | **Year** | **Survey Question** | **Question ID** |
| --- | --- | --- | --- |
| Asthma | 2005–2018 | Has a doctor or other health professional EVER told you that “SC name” had asthma? | CHS.080_00.000 |
| AD | 2005–2018 | DURING THE PAST 12 MONTHS, has “SC name” had any of the following conditions … eczema or any kind of skin allergy? | CHS.115_04.000 |
| ID | 2005–2010 | Has a doctor or health professional ever told you that “SC name” had … mental retardation? | CHS.032_02.000 |
|  | 2011–2018 | Has a doctor or health professional ever told you that “SC name” had … an intellectual disability also known as mental retardation? | CHS.032_02.000 |
| ASD | 2005–2010 | Has a doctor or health professional ever told you that “SC name” had … autism? | CHS.060_06.000 |
|  | 2011–2013 | Has a doctor or health professional ever told you that “SC name” had … autism/autism spectrum disorder? | CHS.061_06.000 |
|  | 2014–2018 | Has a doctor or health professional ever told you that “SC name” had … autism, Asperger’s disorder, pervasive developmental disorder, or autism spectrum disorder? | CHS.032_02.010 |
| ADHD | 2005–2018 | Has a doctor or health professional ever told you that “SC name” had … attention deficit hyperactivity disorder (ADHD) or attention-deficit disorder (ADD)? | CHS.032_01.000 |
| LD | 2005–2018 | Has a representative from a school or a health professional ever told you that “SC name” had … a learning disability? | CHS.312_00.000 |

Data Source: National Health Interview Survey (NHIS), 2005–2018.

AD: atopic dermatitis; ID: intellectual disability; ASD: autism spectrum disorder; ADHD: attention deficit hyperactivity disorder. LD: learning disability.

**eTable 2 Stratified analysis by age for correlations of the T2 diseases with four neurodevelopmental disorders in US children aged 3–17 years in the NHIS, 2005–2018**

| **Outcomes** | **Variables** | **No. of cases/total participants** | | **OR  (95% CI)** | **P** | **No. of cases/total participants** | | **OR  (95% CI)** | **P** | **No. of cases/total participants** | | **OR  (95% CI)** | **P** |
| --- | --- | --- | --- | --- | --- | --- | --- | --- | --- | --- | --- | --- | --- |
|  |  | **Without asthma** | **With asthma** |  |  | **Without AD** | **With AD** |  |  | **Without T2 diseases** | **With T2 diseases** |  |  |
| **ID** |  |  |  |  |  |  |  |  |  |  |  |  |  |
| Age | 3–11y | 92  /5430 | 36  /1289 | 1.11  (0.65, 1.90) | 0.004 | 102  /5824 | 26  /895 | 1.24  (0.74, 2.09) | <0.001 | 76  /4846 | 52  /1873 | 1.12  (0.71, 1.76) | 0.589 |
|  | 12–17y | 86  \|/3522 | 42  \|/1019 | 1.59  (1.02, 2.48) |  | 112  \|/4060 | 16  \|/481 | 1.34  (0.69, 2.57) |  | 75  \|/3223 | 53  \|/1318 | 1.68  (1.10, 2.56) |  |
| **ASD** |  |  |  |  |  |  |  |  |  |  |  |  |  |
| Age | 3–11y | 101  \|/5430 | 44  \|/1289 | 1.68  (1.08, 2.61) | <0.001 | 120  \|/5824 | 25  \|/895 | 1.89  (1.11, 3.22) | <0.001 | 88  \|/4846 | 57  \|/1873 | 1.79  (1.18, 2.71) | 0.151 |
|  | 12–17y | 62  \|/3522 | 20  \|/1019 | 0.89  (0.48, 1.65) |  | 70  \|/4060 | 12  \|/481 | 1.65  (0.75, 3.65) |  | 56  \|/3223 | 26  \|/1318 | 0.98  (0.55, 1.75) |  |
| **ADHD** |  |  |  |  |  |  |  |  |  |  |  |  |  |
| Age | 3–11y | 373  \|/5430 | 190  \|/1289 | 1.90  (1.47, 2.48) | 0.116 | 456  \|/5824 | 107  \|/895 | 1.45  (1.06, 1.99) | 0.026 | 319  \|/4846 | 244  \|/1873 | 1.75  (1.37, 2.23) | 0.993 |
|  | 12–17y | 406  \|/3522 | 218  \|/1019 | 2.14  (1.67, 2.73) |  | 543  \|/4060 | 81  \|/481 | 1.31  (0.92, 1.86) |  | 365  \|/3223 | 259  \|/1318 | 1.91  (1.51, 2.43) |  |
| **LD** |  |  |  |  |  |  |  |  |  |  |  |  |  |
| Age | 3–11y | 465  \|/5430 | 207  \|/1289 | 1.59  (1.27, 2.00) | 0.114 | 541  \|/5824 | 131  \|/895 | 1.66  (1.26, 2.18) | 0.045 | 386  \|/4846 | 286  \|/1873 | 1.73  (1.41, 2.12) | 0.730 |
|  | 12–17y | 428  \|/3522 | 215  \|/1019 | 1.84  (1.40, 2.41) |  | 552  \|/4060 | 91  \|/481 | 1.47  (1.06, 2.02) |  | 378  \|/3223 | 265  \|/1318 | 1.79  (1.40, 2.28) |  |

Data Source: National Health Interview Survey (NHIS), 2005–2018.

OR, odds ratio; AD: atopic dermatitis; T2 diseases: type 2 inflammatory diseases (including asthma or atopic dermatitis here).

ID: intellectual disability; ASD: autism spectrum disorder; ADHD: attention deficit hyperactivity disorder; LD: learning disability.

**eTable 3 Stratified study by sex for correlations of the T2 diseases with four neurodevelopmental disorders in US children aged 3 to 17 years in NHIS, 2005–2018**

| **Outcomes** | **Variables** | **No. of cases/total participants** | | **OR  (95% CI)** | **P** | **No. of cases/total participants** | | **OR  (95% CI)** | **P** | **No. of cases/total participants** | | **OR  (95% CI)** | **P** |
| --- | --- | --- | --- | --- | --- | --- | --- | --- | --- | --- | --- | --- | --- |
|  |  | **Without asthma** | **With asthma** |  |  | **Without AD** | **With AD** |  |  | **Without T2 diseases** | **With T2 diseases** |  |  |
| **ID** |  |  |  |  |  |  |  |  |  |  |  |  |  |
| Sex | Male | 100  /4168 | 54  /1284 | 1.47  (0.95, 2.26) | <0.001 | 128  /4781 | 26  /671 | 1.40  (0.84, 2.33) | 0.123 | 84  /3760 | 70  /1692 | 1.58  (1.03, 2.43) | <0.001 |
|  | Female | 78  /4784 | 24  /1024 | 1.03  (0.57, 1.88) |  | 86  /5103 | 16  /705 | 1.26  (0.65, 2.44) |  | 67  /4309 | 35  /1499 | 1.04  (0.61, 1.77) |  |
| **ASD** |  |  |  |  |  |  |  |  |  |  |  |  |  |
| Sex | Male | 117  /4168 | 47  /1284 | 1.25  (0.82, 1.89) | <0.001 | 141  /4781 | 23  /671 | 1.38  (0.78, 2.44) | 0.056 | 106  /3760 | 58  /1692 | 1.28  (0.86, 1.89) | <0.001 |
|  | Female | 46  /4784 | 17  /1024 | 1.60  (0.75, 3.42) |  | 49  /5103 | 14  /705 | 2.99  (1.35, 6.62) |  | 38  /4309 | 25  /1499 | 1.95  (0.97, 3.91) |  |
| **ADHD** |  |  |  |  |  |  |  |  |  |  |  |  |  |
| Sex | Male | 515  /4168 | 279  /1284 | 1.67  (1.36, 2.06) | <0.001 | 683  /4781 | 111  /671 | 1.25  (0.95, 1.65) | 0.020 | 461  /3760 | 333  /1692 | 1.54  (1.27, 1.88) | <0.001 |
|  | Female | 264  /4784 | 129  /1024 | 2.54  (1.81, 3.58) |  | 316  /5103 | 77  /705 | 1.81  (1.24, 2.62) |  | 223  /4309 | 170  /1499 | 2.39  (1.76, 3.23) |  |
| **LD** |  |  |  |  |  |  |  |  |  |  |  |  |  |
| Sex | Male | 535  /4168 | 267  /1284 | 1.56  (1.27, 1.92) | <0.001 | 665  /4781 | 137  /671 | 1.91  (1.47, 2.48) | 0.957 | 452  /3760 | 350  /1692 | 1.82  (1.51, 2.19) | <0.001 |
|  | Female | 358  /4784 | 155  /1024 | 1.85  (1.35, 2.53) |  | 428  /5103 | 85  /705 | 1.35  (1.01, 1.80) |  | 312  /4309 | 201  /1499 | 1.68  (1.29, 2.19) |  |

Data Source: National Health Interview Survey (NHIS), 2005–2018.

OR, odds ratio; AD: atopic dermatitis; T2 diseases: type 2 inflammatory diseases (including asthma or atopic dermatitis here).

ID: intellectual disability; ASD: autism spectrum disorder; ADHD: attention deficit hyperactivity disorder; LD: learning disability.

**eTable 4 Stratified analysis by race/ethnicity for correlations of the T2 diseases with four neurodevelopmental disorders in US children aged 3–17 years in the NHIS, 2005–2018**

| **Outcomes** | **Variables** | **No. of cases/total participants** | | **OR  (95% CI)** | **P** | **No. of cases/total participants** | | **OR  (95% CI)** | **P** | **No. of cases/total participants** | | **OR  (95% CI)** | **P** |
| --- | --- | --- | --- | --- | --- | --- | --- | --- | --- | --- | --- | --- | --- |
|  |  | **Without asthma** | **With asthma** |  |  | **Without AD** | **With AD** |  |  | **Without T2 diseases** | **With T2 diseases** |  |  |
| **ID** |  |  |  |  |  |  |  |  |  |  |  |  |  |
| Race  /ethnicity | White people | 116  /5917 | 47  /1313 | 1.44  (0.93, 2.22) | <0.001 | 139  /6459 | 24  /771 | 1.47  (0.87, 2.51) | <0.001 | 99  /5384 | 64  /1846 | 1.53  (1.03, 2.29) | <0.001 |
|  | Nonwhite people | 62  /3035 | 31  /995 | 1.15  (0.64, 2.08) |  | 75  /3425 | 18  /605 | 1.21  (0.66, 2.23) |  | 52  /2685 | 41  /1345 | 1.14  (0.68, 1.92) |  |
| **ASD** |  |  |  |  |  |  |  |  |  |  |  |  |  |
| Race  /ethnicity | White people | 110  /5917 | 42  /1313 | 1.27  (0.80, 2.00) | <0.001 | 128  /6459 | 24  /771 | 2.18  (1.26, 3.79) | <0.001 | 95  /5384 | 57  /1846 | 1.62  (1.07, 2.45) | <0.001 |
|  | Nonwhite people | 53  /3035 | 22  /995 | 1.54  (0.85, 2.81) |  | 62  /3425 | 13  /605 | 1.35  (0.62, 2.92) |  | 49  /2685 | 26  /1345 | 1.24  (0.68, 2.25) |  |
| **ADHD** |  |  |  |  |  |  |  |  |  |  |  |  |  |
| Race  /ethnicity | White people | 528  /5917 | 217  /1313 | 1.83  (1.44, 2.32) | <0.001 | 645  /6459 | 100  /771 | 1.26  (0.91, 1.75) | 0.003 | 465  /5384 | 280  /1846 | 1.71  (1.37, 2.15) | <0.001 |
|  | Nonwhite people | 251  /3035 | 191  /995 | 2.34  (1.77, 3.08) |  | 354  /3425 | 88  /605 | 1.74  (1.25, 2.42) |  | 219  /2685 | 223  /1345 | 2.11  (1.63, 2.72) |  |
| **LD** |  |  |  |  |  |  |  |  |  |  |  |  |  |
| Race  /ethnicity | White people | 608  /5917 | 235  /1313 | 1.46  (1.17, 1.81) | 0.002 | 715  /6459 | 128  /771 | 1.56  (1.21, 2.01) | <0.001 | 524  /5384 | 319  /1846 | 1.61  (1.32, 1.95) | <0.001 |
|  | Nonwhite people | 285  /3035 | 187  /995 | 2.14  (1.64, 2.79) |  | 378  /3425 | 94  /605 | 1.79  (1.29, 2.47) |  | 240  /2685 | 232  /1345 | 2.10  (1.64, 2.69) |  |

Data Source: National Health Interview Survey (NHIS), 2005–2018.

OR, odds ratio; AD: atopic dermatitis; T2 diseases: type 2 inflammatory diseases (including asthma or atopic dermatitis here).

ID: intellectual disability; ASD: autism spectrum disorder; ADHD: attention deficit hyperactivity disorder; LD: learning disability.
